# 24‐hour ambulatory blood pressure alterations in patients with Parkinson's disease

**DOI:** 10.1002/brb3.2428

**Published:** 2021-11-28

**Authors:** Liwei Shen, Xiaoli Yang, Wenmei Lu, Weijie Chen, Xiaofei Ye, Danhong Wu

**Affiliations:** ^1^ Department of Neurology Shanghai Fifth People's Hospital, Fudan University Shanghai China; ^2^ Department of Health Statistics Naval Medical University Shanghai China

**Keywords:** 24‐h ambulatory blood pressure, blood pressure variability, circadian blood pressure rhythm, Parkinson's disease

## Abstract

**Objects:**

Abnormal blood pressure (BP) regulation is a feature of autonomic dysfunction in Parkinson's disease (PD) patients. The present study was to analyze the BP alterations by 24‐h ambulatory BP monitoring in PD patients with different disease stages and subtypes.

**Methods:**

32 consecutive patients PD patients and 43 control patients in our hospital from 2017 to 2020 were included. The circadian BP rhythm was divided into three types according to the 24‐h ambulatory BP monitoring. Dipping was defined as an average systolic BP (SBP) reduction during night‐time of 10%–19%. Reverse dipping was defined as an average increase in night‐time SBP values. The differences of the circadian BP rhythm and BP variability (BPV) were analyzed between PD group and the control group, the early PD group and the advanced PD group, as well as the tremor‐dominant group and the nontremor‐dominant group.

**Results:**

There was statistical difference in circadian BP rhythm between PD group and control group (*p* < .05). There were statistical differences in circadian BP rhythm between the early PD group and the advanced PD group (*p* < .05). The mean values of night‐time SBP and diastolic BP (DBP) in the advanced PD group were higher than those in the control group and the early PD group (*p* < .05). The DBP CV in the advanced PD group was higher than that in the control group and the early PD group (*p* < .05). There was no significant difference of circadian BP rhythm, mean BP, and BPV between the tremor‐dominant and the nontremor‐dominant PD group after matching the disease duration.

**Conclusions:**

Reverse dipping was more common in PD patients in this study, especially in the advanced PD patients. 24‐h ambulatory BP monitoring is an important method to evaluate the BP alterations in PD patients. Clinicians should be alert to reverse dipping in PD patients and intervene to prevent serious clinical events.

## Graphical abstract

This study was to evaluate the characteristics of blood pressure (BP) alterations in Parkinson's disease (PD) patients by 24‐h ambulatory BP monitoring. Reverse dipping was more common in PD patients in this study, especially in advanced PD patients. Clinicians should be alert to reverse dipping in PD patients and intervene to prevent serious clinical events.

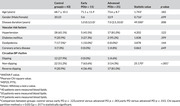

## 1 INTRODUCTION

Parkinson's disease (PD) is common degeneration disease of central nervous system. The clinical features of PD include motor symptoms such as bradykinesia, rigidity, tremor, postural instability, and non‐motor symptoms such as hyposmia, depression, sleep disorders, and autonomic dysfunction. Some non‐motor symptoms can precede the onset of motor symptoms several years. Autonomic dysfunction is a common non‐motor feature in PD patients, whose prevalence is 30%–40% (Duz & Yilmaz, 2020). Although autonomic dysfunction includes different features in PD, cardiovascular autonomic dysfunction is a common feature in PD, which is often characterized by blood pressure abnormalities, such as orthostatic hypotension (OH), supine hypertension, and postprandial hypotension. Since degeneration of the cardiac sympathetic nerve begins in the early stage of PD (Asahina et al., 2013), abnormal blood pressure may appear early in the disease duration. As we know, PD can be divided into two types according to the tremor symptom (Asahina et al., 2013). The nontremor‐dominant PD is associated with more non‐motor symptoms, such as depression, dementia, and autonomic dysfunction than the tremor‐dominant PD (Ejaz et al., 2006). Blood pressure abnormalities may be more common in the nontremor‐dominant PD. Therefore, detection of blood pressure abnormalities may be helpful to diagnose PD in the early motor or premotor stages.

Blood pressure abnormalities lead to a decrease in cerebral perfusion and the characteristic symptoms include dizziness, blurred vision, even fall, and syncope (Espay et al., 2016). Long‐term blood pressure abnormalities can increase the risk of cardiovascular events (Fanciulli et al., 2014). So it is also important to detect the blood pressure abnormalities in order to prevent the development of some serious symptoms.

Although there were some studies detecting the blood pressure abnormalities in PD patients (Hawkes et al., 2010; Hughes et al., 1992; Kanegusuku et al., 2017; Luis & Felix, 2017), few studies have focused on the BP abnormalities of different PD disease stages or subtypes. 24‐h ambulatory blood pressure monitoring (ABPM) is a useful tool to evaluate blood pressure alterations. It can be used to evaluate circadian blood pressure rhythm and blood pressure variability (BPV) with the advantages such as noninvasive, intermittent, etc. This study intended to analyze the blood pressure alterations and BPV in PD patients with different disease stages and subtypes by 24‐h ABPM in order to find potential markers which can be used to help the early diagnosis of autonomic dysfunction and provide basis for the treatment of blood pressure abnormalities.

## 2 METHOD

### 2.1 Study population

The study was approved by the ethical committee of the Shanghai Fifth People's hospital, and informed consent were signed from patients or their representatives. The study was performed in accordance with the Declaration of Helsinki regarding the ethical principles for research involving human subjects.

This study enrolled PD patients who were treated in the Department of Neurology of Shanghai Fifth People's Hospital of Fudan University from January 2017 to June 2020. Patients were included if they met the following criteria: (1) met the UK idiopathic Parkinson's disease brain bank criteria (Mena et al., 2005), (2) measured by 24‐h ABPM. Patients who met the following criteria were excluded: (1) diagnosed with parkinsonism or other extrapyramidal diseases, (2) not measured by 24‐h ABPM. According to Hoehn & Yarh (H&Y) scale, these PD patients were divided into two groups, early stage refers to H&Y 1–2.5 and advanced stage refers to H&Y 3–5. According to the tremor symptom, PD patients were divided into two subtypes: tremor‐dominant group and nontremor‐dominant group.

Patients with benign paroxysmal positional vertigo (BPPV) and chronic subjective dizziness in the hospital at the same time were included as control group. There were no significant differences of the proportion of hypertension, diabetes mellitus, dyslipidemia, and coronary artery disease between PD group and control group.

### 2.2 24‐h ambulatory blood pressure monitoring

The 24‐h ambulatory blood pressure (BP) monitors were produced by A&D Company Limited. Patients were measured BP and heart rate (HR) every 30 min during 6:00 a.m. to 10:00 p.m. and every 60 min during 10:00 p.m. to 6:00 a.m. the next day. The mean blood pressure from 6:00 a.m. to 10:00 p.m. is the mean daytime blood pressure. The mean blood pressure from 10:00 pm to 6:00 a.m. the next day is the mean night‐time blood pressure. We chose the nontremor arm or the less tremor arm of the patient for ABPM.

The dipping pattern was classified according to the recommendations of the European Society of Hypertension (Merola et al., 2018) as follows:

1. Extreme dipping: systolic night‐time blood pressure/systolic daytime blood pressure ≤0.8, corresponding to an average systolic BP (SBP) reduction during night‐time greater or equal to 20%.
2. Normal dipping: systolic night‐time blood pressure/systolic daytime blood pressure >0.8 but ≤0.9, corresponding to an average SBP reduction during night‐time of 10%−19%.
3. Reduced dipping: systolic night‐time blood pressure/systolic daytime blood pressure >0.9 but ≤1.0, corresponding to an average SBP reduction during night‐time of 0%−9%.
4. Reverse dipping: systolic night‐time blood pressure/systolic daytime blood pressure ≥1.0, corresponding to an average increase in night‐time SBP values.

Blood pressure is not constant and there is spontaneous variation in BP. The variation in BP is defined as BPV. Coefficient of variation (CV) is a common indicator of BPV, which is the standard deviation divided by the corresponding mean value. The average real variability (ARV) is a useful indicator for studying the clinical value of BPV proposed by Mena et al., which calculates the average of absolute changes between consecutive BP readings (Milazzo et al., 2018). We used the coefficient of variation (CV) and ARV as indicators of BPV. Calculation formula as follows:

1. CV = Standard deviation/Average value × 100%
2. $\mathrm{ARV}=\frac{1}{N-1}\sum_{k=1}^{N-1}|BP_{k+1}-BP_{k}|$

### 2.3 Statistical analysis

SPSS 22.0 statistical software was used for statistical analysis. The data conforming to normal distribution were expressed as mean ± standard deviation (χ ± s) data. Independent sample *t* test was used between two groups if the continuous variables conformed to the homogeneity of variance. One‐way ANOVA was used for multi‐group comparison, and post hoc test (LSD‐t test) is used for two group's comparison. The data which did not conform to normal distribution was expressed as M(P25, P75). Nonparametric tests was used if the data did not conform to normal distribution. Mann–Whitney *u* test was used for the comparison of two groups, while Kruskal–Wallis test was used for the comparison of multi‐groups. Chi‐square test was used for frequency analysis of classified variables. The Chi‐square partition method was used to compare between group differences and the recalculated test level was 0.05 divided by comparison times. *p* < .05 is statistically significant.

Propensity score matching (PSM) method was used to match the interference of disease duration between groups. We applied PSM module in SPSS 22.0 statistical software to complete PSM analysis. Each PD patient was sequentially given an identification number. The group variables such as PD subtypes were used as group indicator and disease duration was used as predictor. Match tolerance was defined as 0.05. The software output a new dataset after matching. Independent sample *t* test was used to compare continuous variables between two groups after PSM. Chi‐square test is used for frequency analysis of classified variables after PSM. *p* < .05 is statistically significant.

## 3 RESULT

### 3.1 Basic information of selected patients

**FIGURE 1:**
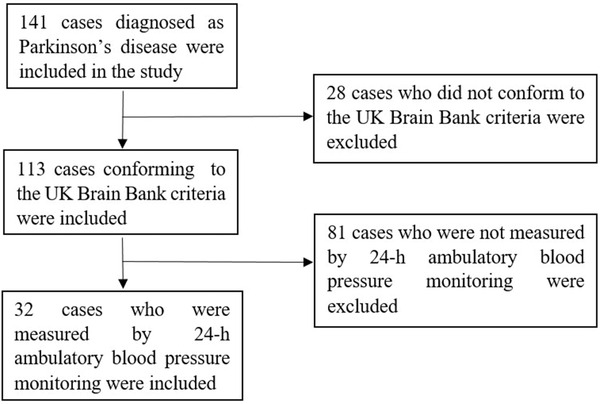
Flowchart of case selection

141 cases of Parkinson's disease were collected. After excluding 28 cases who did not conform to the UK Brain Bank criteria, 81 patients with Parkinson's disease without complete 24‐h ABPM data were excluded, 32 cases with Parkinson's disease were finally included in the study, Figure 1.

17 males and 15 females with an average age of 72.7 ± 9.8 years were included in the PD group. 20 males and 23 females with an average age of 68.7 ± 10.1 years were included in the control group. There was no significant difference of gender, age and the proportion of hypertension, diabetes mellitus, dyslipidemia, coronary artery disease between the two groups (*p* > .05), Table 1.

**TABLE 1:** Demographic and clinical characteristics of the study population

	**Control group (*n* = 43)**	**PD group (*n* = 32)**	**Statistic value**	***p* value**
**Demographic characteristics**				
Age (years)	68.7 ± 10.1	72.7 ± 9.8	1.750^a^	.084
Gender (Male:Female)	20:23	17:15	0.644^b^	.644
Disease duration (years)		5.0 (1.0,10.0)^c^		
H&Y stage		2.9 ± 0.6		
LEDD (mg)		431.3 ± 213.8		
**Vascular risk factors**				
Hypertension	28 (65.1%)	22 (68.8%)	0.109^b^	.808
Diabetes mellitus	9 (20.9%)	5 (15.6%)	0.340^b^	.766
Dyslipidemia	7 (17.5%)^d^	1 (3.4%)^e^	2.013^f^	.156
Coronary artery disease	3 (7.0%)	1 (3.1%)	0.046^f^	.830

*Abbreviation*: LEDD, levodopa equivalent daily dose.

^a^ *t* value.

^b^ Pearson Chi‐square value.

^c^ Median expressed as M(P25, P75).

^d^ 40 patients were measured blood lipids in the control group.

^e^ 29 patients were measured blood lipids in PD group.

^f^ Continuity correction value.

### 3.2 Comparison of circadian BP rhythm, mean BP, and BPV between PD group and control group

The circadian BP rhythm in PD group was significantly different from that in the control group (*p* < .05). The proportion of reverse dipping was the highest in PD group (21 cases, 65.6%), and the proportion of non‐dipping was the highest in the control group (22 cases, 51.2%). The night‐time SBP of PD group was 140.2 ± 26.9 mmHg, which was higher than that of the control group 127.5 ± 21.8 mmHg (*p* < .05), Table 2.

**TABLE 2:** Comparison of circadian BP rhythm, mean BP, and BPV between PD group and control group

	**Control group (*n* = 43)**	**PD group (*n* = 32)**	**Statistic value**	***p* value**
**Circadian BP rhythm**				
Dipping	12 (27.9%)	1 (3.1%)		
Non‐dipping	22 (51.2%)	10 (31.3%)	15.842^a^	<.001
Reverse dipping	9 (20.9%)	21 (65.6%)		
**Mean BP**				
Early morning SBP	129.2 ± 36.5	133.3 ± 33.7	0.501	.618
Early morning DBP	74.6 ± 20.2	71.8 ± 18.2	−0.616	.540
Daytime SBP	134.5 ± 18.6	132.3 ± 21.0	−0.470	.640
Daytime DBP	76.7 ± 9.0	73.0 ± 9.8	−1.713	.091
Night‐time SBP	127.5 ± 21.8	140.2 ± 26.9	2.250	.027
Night‐time DBP	71.4 ± 10.2	76.7 ± 13.5	1.938	.056
24‐h SBP	132.7 ± 18.6	134.4 ± 21.6	0.355	.723
24‐h DBP	75.4 ± 8.7	74.1 ± 10.2	−0.561	.577
**BP variability**				
SBP ARV	15.3 ± 4.1	16.1 ± 4.6	0.770	.444
DBP ARV	9.6 ± 2.9	10.0 ± 3.3	0.507	.613
SBP CV (%)	13.2 ± 4.4	14.3 ± 4.1	1.077	.285
DBP CV (%)	14.4 ± 4.5	15.5 ± 5.1	0.951	.345

*Abbreviations*: ARV, average real variability; BP, blood pressure; CV, coefficient of variation; DBP, diastolic blood pressure (value in mmHg); SBP, systolic blood pressure (value in mmHg).

^a^ Chi‐square value.

### 3.3 Comparison of circadian BP rhythm, mean BP, and BPV between the early PD group, advanced PD group, and the control group

The disease duration in the advanced PD group was longer than that in the early PD group (*p* < .05). There was no significant difference of the circadian BP rhythm between the early PD group and the control group. There was statistical difference of the circadian BP rhythm between the advanced PD group and the early PD group (*p* < .05). The proportion of non‐dipping was the highest (63.6%) in the early PD group, while the proportion of reverse dipping was the highest in the advanced PD group (81.0%). There was statistically significant difference in the circadian BP rhythm between the control group and the advanced PD group (*p* < .05), Table 3. The mean values of night‐time SBP and diastolic BP (DBP) in the advanced PD group were higher than those in the control group and the early PD group (*p* < .05). The DBP CV in the advanced PD group was higher than that in the control group and the early PD group (*p* < .05), but no difference of DBP ARV between the control group and the early PD group, Table 4.

**TABLE 3:** Comparison of circadian BP rhythm between early PD group, advanced PD group, and control group

	**Control group (*n* = 43)**	**Early PD (*n* = 11)**	**Advanced PD (*n* = 21)**	**Statistic value**	***p* value**
Age (years)	68.7 ± 10.1	71.1 ± 11.9	73.6 ± 8.7	1.743^a^	.182
Gender (Male:Female)	20:23	5:6	12:9	0.716^b^	.699
Disease duration (years)		1.0 (0.3,5.0)^c^	7.0 (2.5,10.0)^c^	49.500^d^	.008
**Vascular risk factors**					
Hypertension	28 (65.1%)	5 (45.5%)	17 (81.0%)	4.202	.122
Diabetes mellitus	9 (20.9%)	3 (27.3%)	2 (95.2%)	1.838	.399
Dyslipidemia	7 (17.5%)^e^	1 (10.0%)^f^	0 (0.0%)^g^	3.878	.144
Coronary artery disease	3 (7.0%)	0 (0.0%)	1 (4.8%)	0.863	.649
**Circadian BP rhythm**					
Dipping	12 (27.9%)	0 (0.0%)	1 (4.6%)		
Non‐dipping	22 (51.2%)	7 (63.6%)	3 (14.3%)	25.170^b^	<.001^h^
Reverse dipping	9 (20.9%)	4 (36.4%)	17 (81.0%)		

^a^ ANOVA F value.

^b^ Pearson Chi‐square value.

^c^ M(P25, P75).

^d^ Mann–Whitney *u* test *u* value.

^e^ 40 patients were measured blood lipids.

^f^ 10 patients were measured blood lipids.

^g^ 19 patients were measured blood lipids.

^h^ Comparison between groups: control versus early PD *p* = .123,control versus advanced PD *p* < .001,early PD versus advanced PD *p* = .015, Chi‐square partition method *α* = 0.05/3,*p* < .017 is statistically significant.

**TABLE 4:** Comparison of mean BP and BPV in early PD group and advanced PD group and control group

	**Control group (*n* = 43)**	**Early PD group (*n* = 11)**	**Advanced PD group (*n* = 21)**	***f* value**	***p* value**
**Mean BP**					
Early morning SBP	129.2 ± 36.5	119.5 ± 46.2	140.6 ± 23.1	1.453	.241
Early morning DBP	74.6 ± 20.2	67.1 ± 25.6	74.3 ± 12.9	0.694	.503
Daytime SBP	134.5 ± 18.6	128.9 ± 22.7	134.1 ± 20.3	0.360	.699
Daytime DBP	76.7 ± 9	71.6 ± 10.6	73.8 ± 9.6	1.655	.198
Night‐time SBP	127.5 ± 21.8	127.4 ± 24.1	146.9 ± 26.4	5.175	.008^a^
Night‐time DBP	71.4 ± 10.2	70.3 ± 9.80	80.1 ± 14.2	4.635	0.013^b^
24‐h SBP	132.7 ± 18.6	128.6 ± 22.8	137.4 ± 20.8	0.785	.460
24‐h DBP	75.4 ± 8.7	71.4 ± 10.2	75.6 ± 10.1	0.894	.414
**BP variability**					
SBP ARV	15.3 ± 4.1	15.2 ± 5.6	18.6 ± 4.1	0.636	.532
DBP ARV	9.6 ± 2.9	9.2 ± 3.5	10.4 ± 3.1	0.653	.524
SBP CV (%)	13.2 ± 4.4	12.3 ± 4.3	15.3 ± 3.7	2.448	.094
DBP CV(%)	14.4 ± 4.5	12.1 ± 3.4	17.2 ± 4.9	5.296	.007^c^

*Abbreviations*: ARV, average real variability; BP, blood pressure; CV, coefficient of variation.; DBP, diastolic blood pressure (value in mmHg); SBP, systolic blood pressure (value in mmHg).

^a^ Comparison between groups post hoc *p* value: control versus early PD *p* = .983, control versus advanced PD *p* = .003, early PD versus advanced PD *p* = .028.

^b^ Comparison between groups post hoc *p* value:control versus early PD *p* = .776, control versus advanced PD *p* = .006, early PD versus advanced PD *p* = .024.

^c^ Comparison between groups post hoc *p* value: control versus early PD *p* = .127, control versus advanced PD *p* = .021, early PD versus advanced PD *p* = .003.

In order to adjust the influence of the disease duration between the early and advanced PD groups, we used PSM method to match disease duration, 7 early PD patients and 21 advanced PD patients were included after PSM. There was statistical difference of the circadian BP rhythm between the two groups after PSM (*p* < .05).There was also significant difference of night‐time SBP and DBP, SBP, and DBP CV between the two groups after PSM (*p* < .05), Table 5.

**TABLE 5:** Comparison in early PD group, and advanced PD group after matching disease duration by PSM

	**Early PD group (*n* = 7)**	**Advanced PD group (*n* = 21)**	**Statistic value**	***p* value**
Age (years)	70.7 ± 7.4	73.6 ± 8.7	−0.741	.466
Disease duration (years)	2.0 (0.3,8.0)^a^	7.0 (2.5,10.0)^a^	46.500^b^	.148
**Circadian BP rhythm**				
Dipping	0 (0.0%)	1 (4.8%)	7.615	.009^c^
Non‐dipping	5 (71.4%)	3 (14.3%)		
Reverse dipping	2 (33.3%)	17 (81.0%)		
**Mean BP**				
Early morning SBP	128.1 ± 27.3	138.4 ± 21.1	−1.034	.311
Early morning DBP	71.4 ± 14.1	72.5 ± 13.2	−0.179	.860
Daytime SBP	127.4 ± 25.6	133.2 ± 19.0	−0.641	.527
Daytime DBP	70.0 ± 11.5	72.6 ± 9.8	−0.578	.568
Night‐time SBP	124.4 ± 22.8	145.3 ± 24.9	−1.955	.061
Night‐time DBP	67.3 ± 9.7	78.8 ± 14.6	−1.938	.064
24‐h SBP	126.7 ± 24.9	136.4 ± 19.4	−1.072	.294
24‐h DBP	69.4 ± 10.9	74.4 ± 10.4	−1.075	.292
**BP variability**				
SBP ARV	16.2 ± 6.3	17.0 ± 3.9	−0.385	.703
DBP ARV	9.9 ± 3.8	10.4 ± 3.0	−0.412	.684
SBP CV (%)	12.1 ± 4.9	15.7 ± 3.3	−2.153	.041^d^
DBP CV (%)	11.7 ± 3.4	17.5 ± 4.7	−3.033	.005^d^

*Abbreviations*: ARV, average real variability; BP, blood pressure; CV, coefficient of variation.; DBP, diastolic blood pressure (value in mmHg); SBP, systolic blood pressure (value in mmHg).

^a^ Median expressed as M(P25, P75).

^b^ Mann–Whitney *u* test *u* value.

^c^ Fisher's Exact Test *p* < .05.

^d^ *t* test *p* < .05.

### 3.4 Comparison of circadian BP rhythm, mean BP, and BPV in tremor‐dominant and nontremor‐dominant PD patients

The disease duration of the tremor‐dominant PD patients was longer than that in the nontremor‐dominant PD patients (*p* < .05). There was no significant difference of circadian BP rhythm, mean BP, and BPV between the two groups, Table 6.

**TABLE 6:** Comparison of circadian BP rhythm, mean BP, and BPV in tremor‐dominant and nontremor‐dominant PD patients

	**Control group (*n* = 43)**	**Tremor‐dominant PD (*n* = 20)**	**Nontremor‐dominant PD (*n* = 12)**	**Statistics value**	***p* value**
Age (years)	68.7 ± 10.1	73.1 ± 8.7	72.1 ± 11.9	1.489	.113
Gender (Male:Female)	20:23	12:8	5:7	1.330	.514
Disease duration (years)		7.5 (2.3,10.0)^a^	1.1 (0.3,5.0)^a^	60.500^b^	.019
H&Y stage		3.0 ± 0.6	2.8 ± 0.6	0.996	.327
**Circadian BP rhythm**					
Dipping	12 (27.9%)	1 (5.0%)	0 (0.0%)		
Non‐dipping	22 (51.2%)	7 (35.0%)	3 (25.0%)	17.106	.001^c^, ^d^
Reverse dipping	9 (20.9%)	12 (60.0%)	9 (75.0%)		
**Mean BP**					
Early morning SBP	129.2 ± 36.5	134.8 ± 26.8	130.9 ± 44.2	0.311	.758
Early morning DBP	74.6 ± 20.2	72.6 ± 14.6	70.7 ± 23.7	0.279	.782
Daytime SBP	134.5 ± 18.6	131.2 ± 23.7	135.3 ± 16.1	−0.400	.692
Daytime DBP	76.7 ± 9.0	72.4 ± 10.5	74.1 ±8.9	−0.477	.637
Night‐time SBP	127.5 ± 21.8	137.3 ± 30.9	145.1 ± 18.7	−0.792	.435
Night‐time DBP	71.4 ± 10.2	76.0 ± 15.6	77.9 ± 9.8	−0.392	.698
24‐h SBP	132.7 ± 18.6	132.9 ± 24.8	136.8 ± 15.4	−0.494	.625
24‐h DBP	75.4 ± 8.7	73.5 ± 11.2	75.3 ± 8.6	−0.477	.637
**BP variability**					
SBP ARV	15.3 ± 4.1	16.5 ± 4.6	15.5 ± 4.9	0.548	.588
DBP ARV	9.6 ± 2.9	10.5 ± 3.5	9.1 ± 2.8	1.136	.265
SBP CV (%)	13.2 ± 4.4	14.5 ± 4.2	13.9 ± 4.1	0.349	.730
DBP CV (%)	14.4 ± 4.5	16.0 ± 5.3	14.6 ± 4.7	0.763	.451

*Abbreviations*: ARV, average real variability; BP, blood pressure; CV, coefficient of variation.; DBP, diastolic blood pressure (value in mmHg); SBP, systolic blood pressure (value in mmHg).

^a^ Median expressed as M(P25, P75).

^b^ Mann–Whitney *u* test *u* value.

^c^ Fisher's Exact Test.

^d^ Comparison between groups: control versus tremor‐dominant PD *p* = .006, control versus nontremor‐dominant *p* = .001, tremor‐dominant PD versus nontremor‐dominant PD *p* = .811, Chi‐square partition method *α* = 0.05/3, *p* < .017 is statistically significant.

**TABLE 7:** Comparison of circadian BP rhythm, mean BP, and BPV in tremor‐dominant and nontremor‐dominant PD patients after matching disease duration by PSM

	**Tremor dominant (*n* = 7)**	**Nontremor dominant (*n* = 12)**	**Statistics value**	***p* value**
Age (years)	73.4 ± 6.4	72.1 ± 11.9		
Disease duration (years)	2.0 (0.3,8.0)^a^	1.1 (0.3,5.0)^a^	40.500^b^	.898
**Circadian BP rhythm**				
Dipping	0 (0.0%)	0 (0.0%)	0.642	
Non‐dipping	3 (42.9%)	3 (25.0%)		.617
Reverse dipping	4 (57.1%)	9 (75.0%)		
**Mean BP**				
Early morning SBP	138.1 ± 34.2	130.9 ± 44.2	0.371	.715
Early morning DBP	73.1 ± 19.4	70.7 ± 23.7	0.234	.818
Daytime SBP	137.3 ± 29.9	134.3 ± 16.1	0.248	.810
Daytime DBP	72.6 ± 15.2	74.1 ± 8.9	−0.241	.816
Night‐time SBP	142.3 ± 37.3	145.1 ± 18.7	−0.185	.859
Night‐time DBP	75.0 ± 19.1	77.9 ± 9.8	−0.376	.717
24‐h SBP	138.6 ± 31.8	136.8 ± 15.4	0.136	.895
24‐h DBP	73.3 ± 15.9	75.3 ± 8.6	−0.302	.771
**BP variability**				
SBP ARV	16.4 ± 5.8	15.5 ± 4.9	0.366	.719
DBP ARV	11.2 ± 3.7	9.1 ± 2.8	0.809	.430
SBP CV (%)	13.4 ± 4.7	13.9 ± 4.1	−0.238	.815
DBP CV (%)	13.1 ± 2.6	14.6 ± 4.7	−0.854	.405

*Abbreviations*: ARV, average real variability; BP, blood pressure; CV, coefficient of variation.; DBP, diastolic blood pressure (value in mmHg); SBP, systolic blood pressure (value in mmHg).

^a^ Median expressed as M(P25, P75).

^b^ Mann–Whitney *u* test *u* value.

We used PSM method to match the disease duration.7 tremor‐dominant PD patients and 12 nontremor‐dominant PD patients were included after PSM. There was no significant difference of circadian BP rhythm, mean BP, and BPV between the two groups after PSM, Table 7.

### 3.5 Comparison of risk factors affecting circadian BP rhythm between PD reverse dipping group and non‐dipping group

There was no significant difference of age, disease duration, gender, proportion of hypertension, proportion of diabetes mellitus, and H&Y stage between PD reverse dipping group and non‐dipping group, Table 8.

**TABLE 8:** Comparison of risk factors affecting circadian BP rhythm between PD reverse dipping group and non‐dipping group

	**PD reverse dipping group (*n* = 21)**	**PD non‐dipping group (*n* = 10)**	**Statistics value**	***p* value**
Age (years)	75.0 ± 7.6	67.3 ± 12.4	−2.138	.041
Disease duration (years)	6.0 (1.1,10.0)^a^	2.0 (0.3,8.0)^a^	83.000^b^	.370
Gender (Male:Female)	10:11	6:4	0.416	.519
Hypertension	16 (76.2%)	5 (50.0%)	2.126	.145
Diabetes mellitus	2 (9.5%)	3 (30.0%)	2.100	.147
Dyslipidemia	1 (5.3%)^c^	0 (0.0%)^d^	0.491	.483
Coronary artery disease	1 (4.8%)	0 (0.0%)	0.492	.483
H&Y stage	3.0 ± 0.6	2.6 ± 0.7	−1.821	.079

^a^ Median expressed as M(P25, P75).

^b^ *u* value.

^c^ 19 patients were measured blood lipids.

^d^ 9 patients were measured blood lipids.

## 4 DISCUSSION

This study analyzed the circadian BP rhythm and BPV in PD patients. We found that the constituent ratio of the circadian BP rhythm in PD patients were different from those in the control group (*p* < .001) .The proportion of reverse dipping in PD group was the highest, accounting for 65.6% of all PD patients, while the proportion of non‐dipping in the control group was the highest, accounting for 51.2%. There were some studies focusing on the BP regulation in PD patients whose results were similar to our study. Tsukamoto found 64.9% nocturnal hypertension in patients with PD, Parkinson Disease Dementia (PDD), and Dementia with Lewy Bodies (DLB), while 18.2% nocturnal hypertension was found in the control group (Hawkes et al., 2010). Vallelonga found 75% PD patients with reverse dipping (Hughes et al., 1992). While there were some studies that were different from our study. Schmidt first found that 23 patients with PD and other extrapyramidal diseases had no significant drop or even increase of blood pressure at night, among which the proportion of reverse dipping in PD patients was 22%, while non‐dipping was 48% (Kanegusuku et al., 2017). Sommer found 43% reverse dipping and 45% non‐dipping in 40 PD patients (Luis & Felix, 2017). Fanciulli found 1% reverse dipping and 6% non‐dipping in 16 PD patients (Muller et al., 2011). Arici Duz's result was 42.9% of reverse dipping and 45.7% of non‐dipping in 35 patients with PD (O'Brien et al., 2013). One possible reason of the difference between those studies and our study is the younger average age of PD patients in those studies than the present study. Another possible reason is the small sample size of these studies that produced different results. All of these studies indicated that PD patients presented with a pathological BP regulation. The mechanisms of pathological nocturnal BP regulation include impaired baroreflex activity, inappropriate sympathetic tone while lying supine at night (Orimo et al., 2007).

In this study, the PD group was divided into the early PD group and the advanced PD group. We found the proportion of reverse dipping in the advanced PD group (81.0%) was higher than that in the early PD group (36.4%). Night‐time SBP and DBP in advanced PD group was higher than that in the early PD group and the control group. The seriously impaired autonomic nervous system in the advanced PD patients may contribute to this result.

PD patients can be divided into tremor‐dominant and nontremor‐dominant subtype. In our study, we only found the disease duration of tremor‐dominant PD was longer than nontremor‐dominant PD. Although the nontremor‐dominant PD is associated with more non‐motor symptoms, there were no significant differences of circadian BP rhythm and BPV between the two subtypes in our studies. After matching the disease duration there were still no significant differences between the two groups. Although the result indicated no differences of ambulatory blood pressure between the two subtypes, we should do further research to confirm it with a larger sample size.

In this study, we used CV and ARV to evaluate BPV. We found DBP CV in advanced PD group was higher than that in the early PD group and the control group, SBP CV in advanced PD group was also higher than that in the early PD group after PSM. But there were no significant differences of SBP and DBP ARV between these groups. BPV reflects 24‐h blood pressure fluctuation. An initial increase in BPV is an independent predictor of cardiovascular events (Prodoehl et al., 2013). The indices of BPV include the standard deviation (SD) of 24‐h ambulatory blood pressure, the CV of blood pressure, ARV, and SD_dn_. SD_dn_ is the average of daytime and night‐time SDs weighted by the duration of the daytime and night‐time intervals. SD_dn_ and ARV are less affected by the measuring time during day and night, both of these indices are better than classical indices (SD and CV) (Rajput & Rozdilsky, 1976). Few studies have investigated BPV in patients with PD. Schmidt compared BPV between PD patients and control or other extrapyramidal syndromes, and reported no difference (Kanegusuku et al., 2017); while Kanegusuku found 21 PD patients presented higher daytime systolic/diastolic CV and SD than 21 controls as well as higher systolic/diastolic SD_dn_ and ARV (Rajput & Rozdilsky, 1976).Since the sample size is small in these studies, further research of larger sample size should be carried out to detect the BPV of PD patients.

Circadian BP rhythm can be affected by some other diseases, such as hypertension, diabetes mellitus. We divided PD patients into two groups, reverse dipping group and non‐dipping group and compared the proportion of hypertension, diabetes mellitus, dyslipidemia, and coronary artery disease between the two groups. There was no significant difference of the proportion of those diseases between the two groups.

Normally, the BP gradually decreases after the morning peak, but it remains at a high level during the daytime. At night, especially after falling asleep, the blood pressure decreases further. This pattern changes in pathological conditions. The circadian BP rhythm is regulated by a variety of nervous and humoral factors. The autonomic nervous system, including sympathetic and parasympathetic system, is important in regulating circadian BP rhythm.

Abnormal blood pressure regulation is common in PD patients and can occur at any stages of the disease duration. Abnormal BP regulation in PD includes orthostatic hypotension, supine hypertension, postprandial hypotension. (Schmidt et al., 2009). These abnormal BP regulation in PD patients leads to the loss of normal circadian BP rhythm, with no significant drop in BP at night or an increase in BP compared with daytime. The present study and other studies found that the circadian BP rhythm was abnormal in the PD group, and the proportion of non‐dipping and reverse dipping was higher in the PD group. Schmidt already found that non‐dipping and reverse dipping of PD patients were often accompanied by orthostatic hypotension (Kanegusuku et al., 2017). Recently, researchers found the diagnostic accuracy of reverse dipping in discriminating cardiovascular dysautonomia (area under curve [AUC] 0.791, specificity 84%, sensitivity 69%) was higher than that of bedside blood pressure ascertainment of neurogenic orthostatic hypotension (0.681, 66%, 69%) and supine hypertension (0.641, 78%, 50%) (Sharabi & Goldstein, 2011). It indicates that reverse nocturnal BP dipping is a marker of cardiovascular dysautonomia in Parkinson disease. Therefore, 24‐h blood pressure monitoring is important to assess cardiovascular dysautonomia in PD patients.

Abnormal blood pressure regulation is associated with cardiovascular dysautonomia in PD patients. The pathophysiological mechanism of cardiovascular dysautonomia in PD patients includes cardiac noradrenergic sympathetic denervation, central and peripheral norepinephrine deficiency, and arterial baroreflex failure (Muller et al., 2011). Pathological researches showed α‐synuclein deposition in the sympathetic and parasympathetic plexus of the heart of PD patients (Sommer et al., 2011). Degeneration of the cardiac sympathetic nerve begins in the early disease process of PD (Asahina et al., 2013). The Braak hypothesis suggests that PD patients have Lewy body pathology in autonomic centers and nerves that include the dorsal motor nucleus of the glossopharyngeal and vagal nerves before nigral involvement (Stuebner et al., 2013; Thenganatt & Jankovic, 2014). Some research found that autonomic dysfunction can occur at the early stage of PD, and before the occurrence of typical motor symptoms (Tsukamoto et al., 2013; Vallelonga et al., 2019). Therefore, cardiovascular dysautonomia in PD patients may occur at the prodrome stage of PD.

So clinicians should pay more attention to the blood pressure monitoring and management in PD patients. All the patients with PD should carry out 24‐h ABPM, which is noninvasive and easy to operate and available in the primary hospitals. Clinicians should be alert to PD patients with reverse dipping who may already have cardiovascular dysautonomia. Proper management of BP in PD patients may reduce the risk of syncope, fall, cardiovascular and stroke event.

There were some limitations in the present study. Small sample size may reduce the statistical power and lead to no statistical difference of some observation indicators. We did not compare our result with other parkinsonism such as multiple system atrophy. Other limitations included that our data was only from one medical center and patients were not repeatedly measured by 24‐h ABPM in different periods. In the future, we will do further research to get more valuable results of the BP regulation in PD patients.

## 5 CONCLUSIONS

Reverse dipping was more common in PD patients rather than the control patients in this study, especially in advanced PD patients. 24‐h ambulatory BP monitoring is an important method to evaluate the BP alterations in PD patients. Clinicians should be alert to PD patients with reverse dipping who may already have cardiovascular dysautonomia and pay more attention to the management of blood pressure in PD patients. Abnormal blood pressure regulation in PD patients should be found in time and intervention should be given to prevent serious clinical events.

## CONFLICT OF INTEREST

The authors declare that they have no competing interests.

## AUTHOR CONTRIBUTIONS

Liwei Shen wrote the article. Liwei Shen and Xiaofei Ye analyzed data. Xiaoli Yang, Wenmei Lu, and Weijie Chen were responsible for collecting data. Danhong Wu proofread and reviewed the manuscript. All authors read and approved the final manuscript.

### PEER REVIEW

The peer review history for this article is available at https://publons.com/publon/10.1002/brb3.2428

## Data Availability

I confirm that my article contains a Data Availability Statement even if no data is available (list of sample statements) unless my article type does not require one.
